# Pleurocidin-family cationic antimicrobial peptides are cytolytic for breast carcinoma cells and prevent growth of tumor xenografts

**DOI:** 10.1186/bcr3043

**Published:** 2011-10-24

**Authors:** Ashley L Hilchie, Carolyn D Doucette, Devanand M Pinto, Aleksander Patrzykat, Susan Douglas, David W Hoskin

**Affiliations:** 1 Department of Microbiology & Immunology, Dalhousie University, 5850 College St., Halifax, B3H 4R2, Canada; 2 Department of Pathology, Dalhousie University, 5850 College St., Halifax, B3H 4R2, Canada; 3 Department of Chemistry, Dalhousie University, 6274 Coburg Rd., Halifax, B3H 4R2, Canada; 4 Institute for Marine Biosciences, National Research Council, 1411 Oxford St., Halifax, B3H 3Z1, Canada; 5 Department of Surgery, Dalhousie University, 1276 South Park St., Halifax, B3H 4R2, Canada

## Abstract

**Introduction:**

Cationic antimicrobial peptides (CAPs) defend against microbial pathogens; however, certain CAPs also exhibit anticancer activity. The purpose of this investigation was to determine the effect of the pleurocidin-family CAPs, NRC-03 and NRC-07, on breast cancer cells.

**Methods:**

MTT (3-(4,5-dimethylthiazol-2-yl)2,5-diphenyltetrazolium bromide) and acid phosphatase cell-viability assays were used to assess NRC-03- and NRC-07-mediated killing of breast carcinoma cells. Erythrocyte lysis was determined with hemolysis assay. NRC-03 and NRC-07 binding to breast cancer cells and normal fibroblasts was assessed with fluorescence microscopy by using biotinylated-NRC-03 and -NRC-07. Lactate dehydrogenase-release assays and scanning electron microscopy were used to evaluate the effect of NRC-03 and NRC-07 on the cell membrane. Flow-cytometric analysis of 3,3'-dihexyloxacarbocyanine iodide- and dihydroethidium-stained breast cancer cells was used to evaluate the effects of NRC-03 and NRC-07 on mitochondrial membrane integrity and reactive oxygen species (ROS) production, respectively. Tumoricidal activity of NRC-03 and NRC-07 was evaluated in NOD SCID mice bearing breast cancer xenografts.

**Results:**

NRC-03 and NRC-07 killed breast cancer cells, including drug-resistant variants, and human mammary epithelial cells but showed little or no lysis of human dermal fibroblasts, umbilical vein endothelial cells, or erythrocytes. Sublethal doses of NRC-03 and, to a lesser extent, NRC-07 significantly reduced the median effective concentration (EC_50_) of cisplatin for breast cancer cells. NRC-03 and NRC-07 bound to breast cancer cells but not fibroblasts, suggesting that killing required peptide binding to target cells. NRC-03- and NRC-07-mediated killing of breast cancer cells correlated with expression of several different anionic cell-surface molecules, suggesting that NRC-03 and NRC-07 bind to a variety of negatively-charged cell-surface molecules. NRC-03 and NRC-07 also caused significant and irreversible cell-membrane damage in breast cancer cells but not in fibroblasts. NRC-03- and NRC-07-mediated cell death involved, but did not require, mitochondrial membrane damage and ROS production. Importantly, intratumoral administration of NRC-03 and NRC-07 killed breast cancer cells grown as xenografts in NOD SCID mice.

**Conclusions:**

These findings warrant the development of stable and targeted forms of NRC-03 and/or NRC-07 that might be used alone or in combination with conventional chemotherapeutic drugs for the treatment of breast cancer.

## Introduction

Despite the decline in the incidence and mortality rates of breast cancer from 1990 to 2005, an estimated 192,370 women were expected to be diagnosed with breast cancer in 2009, and 40,170 women were expected to die of the disease, representing nearly 15% of all cancer-related deaths in American women [1]. Although the treatment of breast cancer varies significantly between patients, treatment options typically include surgery, radiotherapy, chemotherapy, endocrine therapies, and/or administration of trastuzumab [2]. Conventional chemotherapeutic drugs indiscriminately target rapidly dividing cells. Consequently, these drugs fail to kill slow-growing or dormant cancer cells and kill healthy cells that are also rapidly growing, which can lead to adverse side-effects without a reduction in tumor burden [3,4]. The development of multidrug-resistant cancer cells that overexpress drug-efflux pumps such as P-glycoprotein further reduce the effectiveness of conventional chemotherapeutic agents [5]. Furthermore, endocrine-based therapies can lead to the development of secondary malignancies [6]. These shortcomings have led to the development of novel drugs such as trastuzumab (Herceptin), which selectively kill breast cancer cells that express the HER2/neu receptor [7]. However, resistance to trastuzumab caused by altered signal-transduction pathways and decreased interactions between trastuzumab and HER2/neu has already been documented [8]. The need, therefore, persists for a new class of anticancer drugs with the ability to kill cancer cells selectively, regardless of their proliferative capacity, reliance on specific signal-transduction pathways, or the presence of multidrug-resistance proteins. In this regard, certain cationic antimicrobial peptides (CAPs) represent a promising supplement or alternative to current anticancer agents.

CAPs are small peptides (typically consisting of < 50 amino acid residues) that function as an important component of the innate immune system [9]. CAPs are predominantly composed of basic and hydrophobic amino acids and are classified as α-helical, β-sheet, loop, or extended peptides based on the secondary structure that they adopt when in contact with biologic membranes [10]. Compared with normal cells, which have zwitterionic lipids in their membranes and are therefore neutral in charge, the outer-membrane leaflet of cancer cells carries a net negative charge because of a greater abundance of phosphatidylserine residues, *O*-sialoglycoproteins, and heparan sulfate proteoglycans [9,11]. Consequently, certain CAPs have been shown to have a 10-fold greater binding affinity for neoplastic cells in comparison with normal cells, making these CAPs selectively cytotoxic for cancer cells [12]. On binding to the cell, the hydrophobic amino acid side chains of the CAP insert into the hydrophobic core of the membrane, granting the CAP access to the cytosol, and/or leading to cytolysis. Increased transmembrane potential, surface area, and membrane fluidity, all of which are associated with neoplastic cells, may also contribute to the selective cytotoxic activity of certain CAPs [9]. Although many CAPs are cytolytic, others, such as bovine lactoferricin, induce apoptosis in human cancer cell lines through a mechanism that involves reactive oxygen species (ROS) production and mitochondrial membrane destabilization [13].

Certain CAPs kill a wide range of human cancer cells, including multidrug-resistant variants, and show activity against primary tumors and metastatic disease without causing undue harm to vital organs [9,14-18]. Furthermore, because the charge of the cell and not its growth rate determines susceptibility to CAP-mediated cytotoxicity, CAPs are also predicted to target slow-growing or dormant cancer cells. Moreover, resistance of cancer cells to cytolytic CAPs is unlikely, because CAPs are attracted to many negatively-charged surface molecules rather than interacting with a unique receptor; to our knowledge, cancer cell resistance to lytic CAPs has never been documented. In addition, intratumoral administration of lytic peptides has been reported to cause T cell-dependent tumor regression in immune-competent mice and protect the animal from tumor rechallenge [19]. Finally, certain CAPs enhance the cytotoxic activity of traditional anticancer drugs that require access to the cytoplasm to exert their cytotoxic effect [20,21]. Taken together, these findings suggest that CAPs with anticancer activity may be a valuable addition to the medical oncologist's armamentarium.

Fish are heavily dependent on their innate immune system for defense against microbial pathogens and therefore harbor a plethora of novel CAPs with potential anticancer activities. In 2003, 20 pleurocidin-like CAPs (NRC-01 to -20) were identified in various Atlantic flounder species and screened for antimicrobial activity [22]. Our initial screen of a subset of these CAPs revealed that NRC-03 and NRC-07 possess anticancer activity. The amino acid sequence of NRC-03 suggests that when in contact with biologic membranes, NRC-03 contains an unstructured cationic amino terminus followed by an α-helical segment that is kinked near the carboxy-terminus because of the presence of two glycine residues. The amino acid sequence of NRC-07 suggests the formation of a complete α-helix under similar conditions. It is noteworthy that other α-helical CAPs, such as melittin, exhibit potent hemolytic activity [23].

The purpose of this investigation was to determine whether NRC-03 and NRC-07 selectively kill breast cancer cells, including drug-resistant breast cancer cells; to examine whether NRC-03 and NRC-07 enhance the cytotoxic activity of traditional chemotherapeutic drugs; to determine the mechanism of action of NRC-03 and NRC-07; and to study *in vivo *activity of NRC-03 and NRC-07 in a xenograft tumor model. Both NRC-03 and NRC-07 were found to kill breast carcinoma cells, including drug-resistant and slow-growing breast cancer cells. NRC-03 and NRC-07 also reduced the EC_50 _of cisplatin, suggesting their possible use in combination therapy. NRC-03- and NRC-07-mediated cell death correlated with peptide binding to anionic surface molecules. Importantly, NRC-03 and NRC-07 killed breast cancer cells that were grown as xenografts in immune-deficient mice. This is the first study to evaluate pleurocidin family CAPs in terms of their cytotoxicity for breast cancer cells, their mechanism of action, and *in vivo *antitumor activity.

## Materials and methods

### Cell culture and conditions

MDA-MB-231 breast cancer cells were obtained from Dr. S. Drover (Memorial University of Newfoundland, St. John's, NL, Canada). MDA-MB-468, T47-D, SKBR3, MCF7, and paclitaxel-resistant MCF7 (MCF7-TX400) breast cancer cells were obtained from Drs. P. Lee, J. Blay, G. Dellaire, and K. Goralski, respectively (Dalhousie University, Halifax, NS, Canada). 4T1 mouse mammary carcinoma cells were obtained from Dr. D. Waisman (Dalhousie University). Human erythrocytes were obtained from Dr. R. Duncan (Dalhousie University). L cells (immortalized mouse fibroblasts), gro2C cells (heparan sulfate proteoglycan-deficient L cells), and sog9 cells (heparan and chondroitin sulfate proteoglycan-deficient L cells) [24,25], as well as ATG5^+/+ ^and ATG5^-/- ^mouse embryo fibroblasts [26] were obtained from Dr. C. MacCormick (Dalhousie University). All cells were maintained at 37°C in a 5% or 10% CO_2 _humidified atmosphere in RPMI 1640 or DMEM medium (Sigma-Aldrich Canada, Oakville, ON, Canada), respectively. All media were supplemented with 100 U/ml penicillin, 100 μg/ml streptomycin, 2 m*M *L-glutamine, 5 m*M *HEPES (pH 7.4), and 10% heat-inactivated fetal bovine serum (FBS) (Invitrogen, Burlington, ON, Canada). Stock flasks were passaged as required to maintain optimal cell growth and were routinely confirmed to be free from Mycoplasma contamination; however, none of the cell lines has been authenticated. Primary cultures of human umbilical vein endothelial cells (HUVECs), human mammary epithelial cells (HMECs), and human dermal fibroblasts were obtained from Lonza Inc. and maintained in Clonetics EGM-2, MEGM, and FGM-2, respectively, at 37°C in a 5% CO_2 _humidified atmosphere for a maximum of six passages.

### Reagents

NRC-03 (amino acid sequence: GRRKRKWLRRIGKGVKIIGGAALDHL-NH_2_) and NRC-07 (RWGKWFKKATHVGKHVGKAALTAYL-NH_2_) were synthesized by Dalton Pharma Services (Toronto, ON, Canada) or American Peptide Company (Sunnyvale, CA, USA). NRC-13 (GWRTLLKKAEVKTVGKLALKHYL-NH_2_), biotinylated-NRC-03, and biotinylated-NRC-07 were synthesized by Dalton Pharma Services. Peptides were biotinylated at the N-terminus and were > 95% pure. Lyophilized peptides were reconstituted in serum-free DMEM. All experiments were conducted in medium containing 2.5% FBS to limit peptide degradation by serum proteases. Reduced glutathione (GSH), 3-(4,5-dimethylthiazol-2-yl)2,5-diphenyltetrazolium bromide (MTT), paclitaxel, cisplatin, triethylammonium bicarbonate buffer, trifluoroacetic acid, heparan sulfate sodium salt, chondroitin sulfate sodium salt, phosphatase assay substrate, and crystal violet were purchased from Sigma-Aldrich (Oakville, ON, Canada). Sequencing-grade trypsin was obtained from Promega (Madison, WI, USA). Matrix-assisted laser desorption ionization-time of flight (MALDI-TOF) matrix solution was from Agilent Technologies (Palo Alto, CA, USA). Avidin-conjugated horseradish peroxidase (HRP) was purchased from BD Biosciences (San Jose, CA, USA). Hank's balanced salt solution (HBSS) was obtained from Invitrogen. Boc-D-FMK (pan-caspase inhibitor) was purchased from EMD Biosciences (San Diego, CA, USA). Streptavidin-conjugated Texas Red fluorophore was purchased from Jackson Immunoresearch Laboratories (West Grove, PA, USA). *O*-sialoglycoprotein endopeptidase (OSGE) was obtained from Cedarlane Laboratories (Hornby, ON, Canada). Dihydroethidium (DHE), 3,3'-dihexyloxacarbocyanine iodide (DiOC_6_), and Alexa Fluor 488-conjugated phalloidin were purchased from Molecular Probes (Eugene, OR, USA). Mouse anti-cytochrome *c *monoclonal antibody (mAb) was from Upstate Biotechnology (Charlottesville, VA, USA), and mouse anti-mitochondrial Hsp70 mAb was obtained from Affinity BioReagents (Golden, CO, USA). HRP-conjugated goat anti-mouse IgG was purchased from Santa Cruz Biotechnology (Santa Cruz, CA, USA). Fluorescein isothiocyanate (FITC)-conjugated anti-mouse IgG was from eBioscience (San Diego, CA, USA).

### Animals

Adult (6- to 7-week old) female NOD SCID mice purchased from Charles River Canada (Lasalle, QC, Canada) were housed in the Carleton Animal Care Facility and were maintained on a diet of sterilized rodent chow and water *ad libitum*. Animal use was approved by the Dalhousie University Committee on Laboratory Animals and was in accordance with Canadian Council of Animal Care guidelines.

### MTT assay

Breast cancer cell viability was determined by using the MTT assay. In brief, 2 × 10^4 ^breast cancer cells were plated, in quadruplicate, in 96-well flat-bottom tissue-culture plates (Sarstedt, St. Leonard, QC, Canada). After 24-hour culture to promote cellular adhesion, cells were cultured under the indicated conditions for an additional 4 or 24 hours. MTT (100 μg) was added to the culture for the final 2 hours, and the formazan crystals were subsequently solubilized in DMSO (100 μl/well). Absorbance (490 nm) was measured by using a Bio-Tek microplate reader (Bio-Tek Instruments, Winooski, VT, USA). Percentage cytotoxicity was calculated by the formula [1-*E/C*] × 100, where *E *and *C *denote the optical density of peptide- and medium-treated cells, respectively.

### Acid phosphatase assay

The acid phosphatase assay was used instead of the MTT assay to compare NRC-03 and NRC-07 killing of paclitaxel-resistant MCF7-TX400 to wild-type MCF7 cells because drug-efflux pumps interfere with the reduction of MTT [27]. In brief, cells cultured as described for the MTT assay were thoroughly washed with PBS and incubated in 0.1 ml assay buffer (0.1 *M *sodium acetate, 0.1% Triton X-100 (vol/vol), 4 mg/ml phosphatase substrate) for 90 minutes. The reaction was stopped by the addition of 10 μl 1N NaOH. The optical density (405 nm) was measured, and the percentage cytotoxicity was calculated as described for the MTT assay.

### Hemolysis assay

The hemolytic activity of NRC-03 and NRC-07 was determined by culturing human erythrocytes (5% (vol/vol) in PBS) in the presence or absence of the indicated concentrations of NRC-03 and NRC-07 for 8 hours in 96-well round-bottomed tissue-culture plates (Sarstedt). Maximum hemolysis was achieved by combining erythrocytes with an equal volume of water. Erythrocytes were then pelleted (1,400 *g*), and supernatants were transferred to 96-well flat-bottomed tissue-culture plates for analysis. Absorbance (490 nm) was measured, and the percentage hemolysis was calculated by using the equation ((*E/S*)/(*M/S*)) × 100, where *E *and *S *and *M *denote experimental, spontaneous, and maximal hemolysis, respectively.

### Peptide-binding assay

MDA-MB-231 cells and human dermal fibroblasts (8 × 10^5 ^cells) plated in six-well flat-bottom tissue-culture plates containing sterile coverslips were incubated for 24 hours to promote cell adhesion to the coverslips. Adherent cells were cultured for 10 minutes in the presence or absence of 50 μ*M *biotinylated-NRC-03 or -NRC-07. Cells on the coverslips were then fixed with paraformaldehyde (4% (wt/vol) in PBS), washed extensively with PBS, and exposed to Texas Red-conjugated streptavidin (1:1,000) for 45 minutes. After extensive washing with PBS, the coverslips were mounted on slides with Dako fluorescent mounting medium (Dako Canada, Mississauga, ON, Canada). Cells were visualized with phase and fluorescent microscopy at ×400 magnification. Fluorescence per cell was determined by using NIS-Elements software (Nikon Canada, Mississauga, ON, Canada).

### Solid-phase heparan sulfate- and chondroitin sulfate-binding assays

Binding of biotinylated-NRC-03 and biotinylated-NRC-07 to plastic-immobilized heparan sulfate or chondroitin sulfate was determined by using a modification of a previously described protocol [28]. In brief, 10 μg/ml heparan sulfate or chondroitan sulfate in 15 m*M *Na_2_CO_3 _and 35 m*M *NaHCO_3 _buffer (pH 9.2) were incubated overnight in EIA plates at 4°C. Plates were washed with PBS, blocked for 2 hours with 10% FBS, washed again with PBS, and incubated for 2 hours at 23°C in the presence or absence of 50 μ*M *biotinylated-NRC-03 or biotinylated-NRC-07. Plates were then washed with PBS, incubated with avidin-HRP (1:1,000 in PBS) for 1 h, washed with PBS, and incubated with TMB substrate solution. The reaction was stopped by the addition of 0.3 *M *H_2_SO_4_. Peptide binding to heparan sulfate or chondroitin sulfate was determined by measuring absorbance at 450 nm.

### Scanning electron microscopy

MDA-MB-231 cells and human dermal fibroblasts (2 × 10^5 ^cells) were plated in 24-well flat-bottom tissue-culture plates (Sarstedt) containing sterile circular coverslips and incubated overnight to promote cellular adhesion. Cells on coverslips were cultured in the presence or absence of 25 or 50 μ*M *NRC-03, NRC-07, or NRC-13 for 10 or 30 minutes. The cells were washed with 0.1 *M *sodium cacodylate and fixed with glutaraldehyde (2.5% (vol/vol) in sodium cacodylate) for 2 hours. The cells were washed again, fixed with osmium tetroxide (1% (wt/vol) in sodium cacodylate) for 30 minutes, washed again, and dehydrated in increasing concentrations of ethanol until a final concentration of 100% ethanol was achieved. The samples were subsequently dried to their critical point by using a Polaron E3000 Critical Point Dryer (Quorum Technologies, Guelph, ON, Canada), mounted onto stubs, and coated with gold by using a Polaron SC7620 Mini Sputter Coater (Quorum Technologies). The cells were then viewed at the Institute for Research in Materials (Dalhousie University) on a Hitachi S4700 scanning electron microscope (Hitachi High Technologies, Rexdale, ON, Canada) at ×7,000 and ×40,000 magnification.

### Lactate dehydrogenase (LDH)-release assay

The LDH-release assay (CytoTox 96 Non-Radioactive Cytotoxicity Assay; Promega Corporation, Madison, WI, USA) was used per the manufacturer's instructions to quantify cytolysis and to validate cell-viability measurements obtained with MTT assay. Complete cytolysis was achieved by repeated freeze/thaw cycles. Absorbance (490 nm) was used to calculate cytolysis by using the equation ((*E/S*)/(*M/S*)) × 100, where *E *and *S *and *M *denote experimental, spontaneous, and maximal release of LDH, respectively.

### Measurement of mitochondrial transmembrane potential and ROS production

Flow-cytometric analysis of MDA-MB-231 cells stained with DiOC_6 _or DHE was used to measure changes in mitochondrial transmembrane potential and ROS production, respectively. In brief, 5 × 10^5 ^MDA-MB-231 cells were cultured in the presence or absence of 50 μ*M *NRC-03 or NRC-07 for 30 minutes, and then exposed to DHE (2.5 μ*M*) or DiOC_6 _(40 n*M*) 15 minutes before analysis with a FACS Calibur flow cytometer (BD Biosciences, San Jose, CA, USA).

### Mitochondria isolation and Western blotting

Mitochondria were isolated from MDA-MB-231 cells, as previously described [29], and treated with or without 50 μ*M *NRC-03 or NRC-07 for 10 minutes. Mitochondria-free supernatant (cytosolic fraction) was then collected by centrifugation at 12,000 *g *for 10 minutes, and the mitochondria pellet was lysed with ice-cold lysis buffer (50 m*M *Tris (pH 7.5), 150 m*M *NaCl, 50 m*M *Na_2_HPO_4_, 0.25% sodium deoxycholate (wt/vol), 0.1% Nonidet P-40 (vol/vol), 100 μ*M *Na_3_VO_4_, 10 m*M *NaF, 5 m*M *EDTA, and 5 m*M *EGTA) containing freshly added protease inhibitors (final concentration: 1 m*M *phenylmethylsulfonyl fluoride, 10 μg/ml aprotinin, 5 μg/ml leupeptin, 10 μ*M *phenylarsine oxide, 1 m*M *dithiothreitol, and 5 μg/ml pepstatin). Supernatants containing proteins released by lysed mitochondria were collected by centrifugation at 12,000 *g*. Protein concentrations in cytosolic and mitochondrial fractions were determined with the Bradford assay (Bio-Rad Laboratories), and equal amounts of protein (5 μg) were resolved on a 12% SDS-polyacrylamide gel. Proteins were transferred to nitrocellulose membranes, and the resulting blots were blocked for 1 hour in TBS-Tween (0.25 *M *Tris (pH 7.5), 150 m*M *NaCl, 0.2% Tween-20 (vol/vol)) containing 5% powdered skim milk (wt/vol) and probed overnight with the desired primary antibody (1:500). Blots were then washed extensively with TBS-Tween and probed for 1 hour with HRP-conjugated goat anti-mouse IgG (1:1,000). After additional washes, cytochrome *c *or mitochondrial Hsp70 was visualized by using an enhanced chemiluminescence detection system (Bio-Rad Laboratories).

### Confocal microscopy

NRC-03 and NRC-07 subcellular localization was determined by confocal microscopy. In brief, MDA-MB-231 cells (4 × 10^5^) were seeded in six-well flat-bottom tissue-culture plates containing sterile coverslips and were incubated overnight to promote cellular adherence. Cells were then cultured in the presence or absence of biotinylated-NRC-03 or biotinylated-NRC-07 for 30 seconds, washed with PBS, fixed with 4% paraformaldehyde, and washed again with PBS. Cells were then stained with 5 U Alexa Fluor 488 phalloidin (in 0.1% Triton-X 100), TO-PRO-3 iodide (1:1,000 in PBS), or anti-mitochondrial Hsp70 mAb (1:50 in PBS) and FITC-conjugated anti-mouse IgG (1:500 in PBS) for 20 minutes, 10 minutes, and 1 hour, respectively. Stained cells were washed with PBS, incubated in Texas Red-conjugated streptavidin (1.54 μg/ml in PBS) for 45 minutes, washed with PBS, dried, and mounted onto slides by using Dako fluorescent mounting medium. Images (×1,000) were acquired by using an LSM-510 META laser scanning confocal microscope (Carl Zeiss Canada Ltd., Toronto, ON, Canada).

### TUNEL staining

DNA fragmentation was detected by using the TUNEL assay according to the manufacturer's instructions (Roche Diagnostics, Laval, QC, Canada). In brief, MDA-MB-231 breast cancer cells (4 × 10^5^) were seeded into six-well flat-bottom tissue-culture plates containing sterile coverslips, and were incubated overnight to promote cellular adherence. Cells were incubated in the presence or absence of NRC-03 or NRC-07 for 30 minutes, washed with PBS, fixed with 4% paraformaldehyde, washed again with PBS, and incubated at 4°C for 2 minutes in permeabilization solution (0.1% Triton X-100 (vol/vol) in 0.1% sodium citrate (wt/vol)). Cells were then washed with PBS, dried, and stained with TUNEL reaction mixture for 1 hour at 37°C. Stained samples were washed with PBS, dried, mounted on slides by using Dako fluorescent mounting medium, and visualized under visible and UV light (×400).

### Mass spectroscopy

NRC-03 and NRC-07 reconstituted in 50 m*M *TEAB buffer were incubated in the presence or absence of trypsin (2 μg) overnight at 37°C, dried, reconstituted in 0.1% TFA, and diluted 1:1 in Matrix Solution. Samples (500 ng) were spotted on a MALDI plate, dried, and analyzed with a MALDI-TOF mass spectrometer (Waters Corp., Milford, MA, USA).

#### Breast cancer xenografts

NOD SCID mice were engrafted with 5 × 10^6 ^MDA-MB-231 cells by subcutaneous injection in one hind flank. Tumor volume was monitored every other day by using the equation (L*P^2^)/2, where *L *and *P *denote the longest diameter and the diameter perpendicular to the longest diameter, respectively. Once the tumors reached a volume greater than 120 mm^3 ^(approximately 33 days after tumor cell implantation), mice were randomized into groups of three and administered 20 μl of the HBSS vehicle or 0.5 mg NRC-03 or NRC-07 in 20 μl of HBSS by intratumoral injection, beginning on day 1 and repeated on days 3 and 5. Tumor-bearing mice were killed 1 week after the last injection. Tumors were excised, photographed, sectioned, and stained with hematoxylin and eosin. Stained tumor sections were visualized under brightfield microscopy (×400 magnification). The experiment was conducted three times.

### Statistical analysis

All data were analyzed by using the unpaired Student *t *test, or one-way analysis of variance with the Bonferroni multiple comparisons test, as appropriate.

## Results

### NRC-03 and NRC-07 kill breast cancer cells and enhance the efficacy of chemotherapeutic drugs

**Figure 1:**
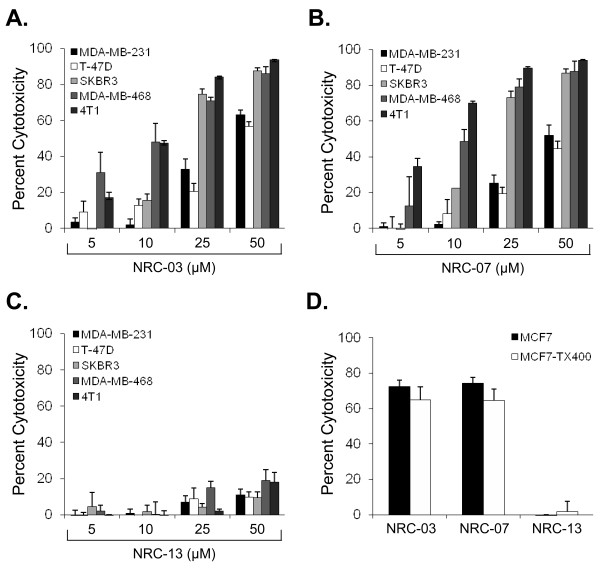
**NRC-03 and NRC-07 are cytolytic for breast cancer cells, including chemoresistant variants**. T-47D, MDA-MB-231, MCF7, SKBR3, and MDA-MB-468 breast cancer cells and 4T1 mouse mammary carcinoma cells were exposed to medium alone or the indicated concentrations of **(a) **NRC-03, **(b) **NRC-07, or **(c) **NRC-13. Cell viability was determined by MTT assay after 24 hours. Data shown are statistically significant by ANOVA (*p *< 0.005). **(d) **MCF7 or paclitaxel-resistant MCF7-TX400 cells were cultured in the presence or absence of 50 μ*M *NRC-03, NRC-07, or NRC-13 for 24 hours. Percentage cytotoxicity relative to cells grown in medium alone was determined with acid phosphatase assay because of the interference of drug-efflux pumps with the reduction of MTT [27]. Cytotoxicity in cultures of NRC-03-, NRC-07, or NRC-13-treated MCF7 cells versus MCF7-TX400 cells was not statistically significant by the Student *t *test (*p *> 0.05). All data shown represent the mean of at least three independent experiments ± SEM.

MTT assays showed that NRC-03 and NRC-07 killed T47-D, MDA-MB-231, MCF7, SKBR3, and MDA-MB-468 breast cancer cells, as well as 4T1 mouse mammary carcinoma cells to a similar extent and in a dose-dependent manner (Figure 1a, b). SKBR3, MDA-MB-468, and 4T1 cells were most susceptible to NRC-03 (75% ± 3%, 86% ± 7%, and 94% ± 1% cytotoxicity, respectively, at 50 μ*M*) and NRC-07 (87% ± 2%, 88% ± 10%, and 94% ± 1% cytotoxicity, respectively, at 50 μ*M*). In contrast, T-47D, MDA-MB-231, and MCF7 cells required 2.5- to 10-fold more NRC-03 and NRC-07 to cause significant cytotoxicity. Nevertheless, MDA-MB-231 cells were chosen as the representative cell line for the remainder of this investigation because these breast cancer cells were susceptible to killing by NRC-03 and NRC-07 and could be grown as xenografts in immune-deficient NOD SCID mice. It is important to note that NRC-13, a noncytotoxic CAP that was used as a control peptide, did not substantially affect the viability of breast cancer cells or mouse mammary carcinoma cells (Figure 1c).

NRC-03-induced cytotoxicity for MDA-MB-231 cells was reduced in the presence of increasing concentrations of FBS (Additional file 1a), suggesting neutralization by anionic serum components and/or susceptibility of the peptide to degradation by proteases. NRC-07-induced cytotoxicity was also diminished by FBS. Similar results were obtained with other breast cancer cell lines (data not shown). Furthermore, mass spectroscopic analysis of trypsin-treated NRC-03 and NRC-07 revealed that both CAPs were extremely sensitive to trypsin-mediated degradation (Additional file 1b).

An acid phosphatase cell-viability assay was used to determine whether NRC-03 and NRC-07 were able to kill drug-resistant breast cancer cells because drug-efflux pumps interfere with the MTT assay [27]. MCF7-TX400 cells are resistant to paclitaxel and express 2.6-fold more P-glycoprotein than do parental cells (data not shown). Figure 1d shows that MCF7 and MCF7-TX400 cells were equally susceptible to 50 μ*M *NRC-03 or NRC-07. As expected, neither MCF7 nor MCF7-TX400 cells were killed by the control peptide NRC-13.

**Figure 2:**
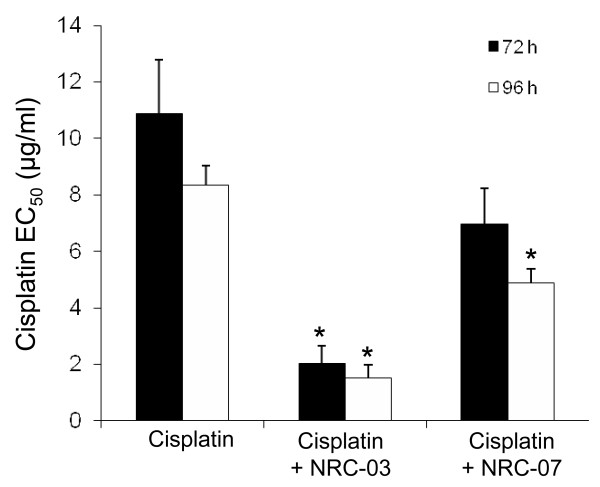
**NRC-03 and NRC-07 enhance cisplatin-mediated cytotoxicity**. MDA-MB-231 cells were cultured in increasing concentrations of cisplatin in the absence or presence of 10 μ*M *NRC-03 or NRC-07. Cell viability, which was used to determine the EC_50 _of cisplatin, was determined with MTT assay after 72 and 96 hours. The EC_50 _of NRC-03 and NRC-07 for MDA-MB-231 cells was determined to be 18.7 ± 2.9 and 18.5 ± 0.9, respectively, across multiple experiments. Data are statistically significant by the Bonferroni multiple comparisons test in comparison with cisplatin-treated cells; *p < 0.05. All data shown are the mean of at least three independent experiments ± SEM.

To determine whether NRC-03 and/or NRC-07 can sensitize breast cancer cells to chemotherapeutic drugs, a sublethal concentration (10 μ*M*) of NRC-03 or NRC-07 was added to MDA-MB-231 cells 20 minutes before their exposure to increasing concentrations of cisplatin (0 to 16 μg/ml). NRC-03 pretreatment reduced the EC_50 _of cisplatin by 5.5-fold after 72 and 96 hours, whereas NRC-07 reduced the EC_50 _of cisplatin by only 1.6- and 1.7-fold after 72 and 96 hours, respectively (Figure 2). Although not shown here, NRC-03 also enhanced killing of MDA-MB-231 cells by docetaxel. NRC-03 is therefore more effective than NRC-07 as a chemosensitizing agent.

Certain CAPs are selectively cytotoxic for cancer cells, whereas other CAPs indiscriminately kill healthy and neoplastic eukaryotic cells [9]. Neither NRC-03 nor NRC-07 killed primary cultures of human dermal fibroblasts or HUVECs at the concentrations that were strongly cytotoxic for MDA-MB-231 breast cancer cells (Table 1). In addition, neither NRC-03 nor NRC-07 exhibited hemolytic activity. However, both NRC-03 and NRC-07 showed substantial cytotoxicity for primary cultures of HMECs, albeit less than was observed with breast cancer cells. Nevertheless, this finding suggests that systemic treatment with NRC-03 and NRC-07 may have adverse consequences *in vivo*.

**Table 1:** Cytotoxic activity of NRC-03 and NRC-07 against normal human cells in comparison to breast cancer cells

	% Cytotoxicity^a^				% Hemolysis^b^
Treatment	HMECs	Fibroblasts	HUVECs	MDA-MB-231	Erythrocytes
25 μ*M *NRC-03	17 ± 5	0 ± 2	3 ± 3	46 ± 6	2 ± 1
50 μ*M *NRC-03	46 ± 3	2 ± 1	19 ± 2	74 ± 4	3 ± 2
25 μ*M *NRC-07	20 ± 4	1 ± 1	7 ± 1	29 ± 8	1 ± 1
50 μ*M *NRC-07	47 ± 9	0 ± 3	17 ± 8	62 ± 5	1 ± 1

^a, b^Mean cytotoxic and hemolytic activity ± SEM (*n *= 3) of NRC-03 or NRC-07 against cultures of normal primary human cells and MDA-MB-231 breast cancer cells, as well as erythrocytes, was determined as described in the Methods section after 24 hours (HMECs, fibroblasts, HUVECs, and erythrocytes) and 8 hours (MDA-MB-231) of exposure to the peptide.

### NRC-03 and NRC-07 interact with negatively-charged cell-surface structures on breast cancer cells

**Figure 3:**
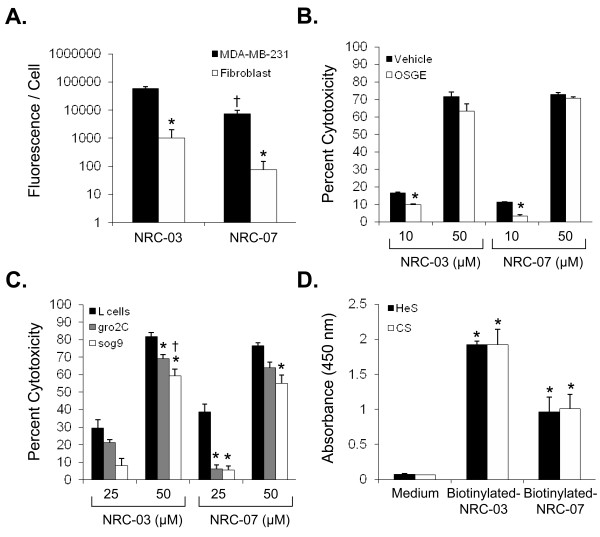
**NRC-03 and NRC-07 bind to anionic structures on breast cancer cells**. **(a) **MDA-MB-231 breast cancer cells or normal dermal fibroblasts were cultured in the absence or presence of 50 μ*M *biotinylated-NRC-03 or -NRC-07 for 10 minutes, stained with Texas Red-streptavidin, and visualized by fluorescence microscopy. Peptide binding was quantified by using NIS-Elements software. Statistical significance was determined with the Bonferroni multiple comparisons test; *p < 0.05 relative to NRC-03- and NRC-07-treated MDA-MB-231 cells, and †*p *< 0.05 relative to NRC-03-treated MDA-MB-231 cells. **(b) **MDA-MB-231 cells were incubated in the absence or presence of *O*-sialoglycoprotein endopeptidease (OSGE) for 30 minutes and then cultured in the absence or presence of 50 μ*M *NRC-03 or NRC-07. Cell viability was determined with the MTT assay after 24 hours. Statistical significance was determined with the Bonferroni multiple comparisons test; *p < 0.05 relative to vehicle-treated cells. **(c) **Wild-type L cells, gro2C cells (heparan sulfate proteoglycan-deficient L cells), or sog9 cells (heparan sulfate proteoglycan- and chondroitin sulfate proteoglycan-deficient L cells) were cultured in the absence or presence of the indicated concentrations of NRC-03 and NRC-07. Cell viability was determined with the MTT assay after 24 hours. Statistical significance was determined with the Bonferroni multiple comparisons test; *p < 0.05 relative to L cells; †*p *< 0.05 relative to gro2C cells. **(d) **Biotinylated-NRC-03 and biotinylated-NRC-07 binding to heparan sulfate (HeS) and chondroitin sulfate (CS) proteoglycans was determined by using the solid-phase heparan sulfate- and chondroitin sulfate-binding assays. Data shown are significant by the Bonferroni multiple comparisons test; *p < 0.05 in comparison with the medium control. All data shown are the mean of three independent experiments ± SEM.

Preferential binding of NRC-03 and NRC-07 to breast cancer cells was assessed with fluorescent microscopy analysis of MDA-MB-231 cells and normal human dermal fibroblasts that were exposed for 10 minutes to biotinylated-NRC-03 and biotinylated-NRC-07, which had cytotoxic activity equivalent to native NRC-03 and NRC-07 (data not shown). Figure 3a shows 56- and 98-fold greater binding of NRC-03 and NRC-07 to MDA-MB-231 cells, respectively, than to fibroblasts. Interestingly, NRC-03 had an eightfold greater affinity for breast cancer cells than did equivalent concentrations of NRC-07, even though NRC-03 and NRC-07 had equivalent cytotoxic activity (Figure 1a).

The outer-membrane leaflet of cancer cells is negatively-charged because of an abundance of anionic molecules, whereas healthy cells are neutral in charge [9,18]. In comparison to MDA-MB-231 cells with intact *O*-sialoglycoproteins, MDA-MB-231 cells that had their *O*-sialoglycoproteins removed by *O*-sialoglycoprotein endopeptidase were only slightly less sensitive to the cytotoxic action of NRC-03 or NRC-07 (Figure 3b), suggesting little if any role for *O*-sialoglycoproteins as major ligands for these CAPs. Because heparan sulfate and chondroitin sulfate proteoglycans are anionic molecules that are often overexpressed on neoplastic cells [11,30], we compared the cytotoxic activity of NRC-03 and NRC-07 against native L cells with L cells that lack heparan sulfate proteoglycan (gro2C cells), and L cells that lack both heparan and chondroitin sulfate proteoglycans (sog9 cells). Gro2C and sog9 cells were less susceptible than native L cells to killing by NRC-03 and NRC-07 (Figure 3c). Sog9 cells showed the most resistance to peptide-mediated cytotoxicity, suggesting that both heparan sulfate and chondroitin sulfate proteoglycans interact with NRC-03 and NRC-07. A solid-phase binding assay confirmed that both NRC-03 and NRC-07 were able to bind immobilized heparan sulfate and chondroitin sulfate proteoglycans (Figure 3d).

### NRC-03 and NRC-07 cause breast cancer cell-membrane damage

**Figure 4:**
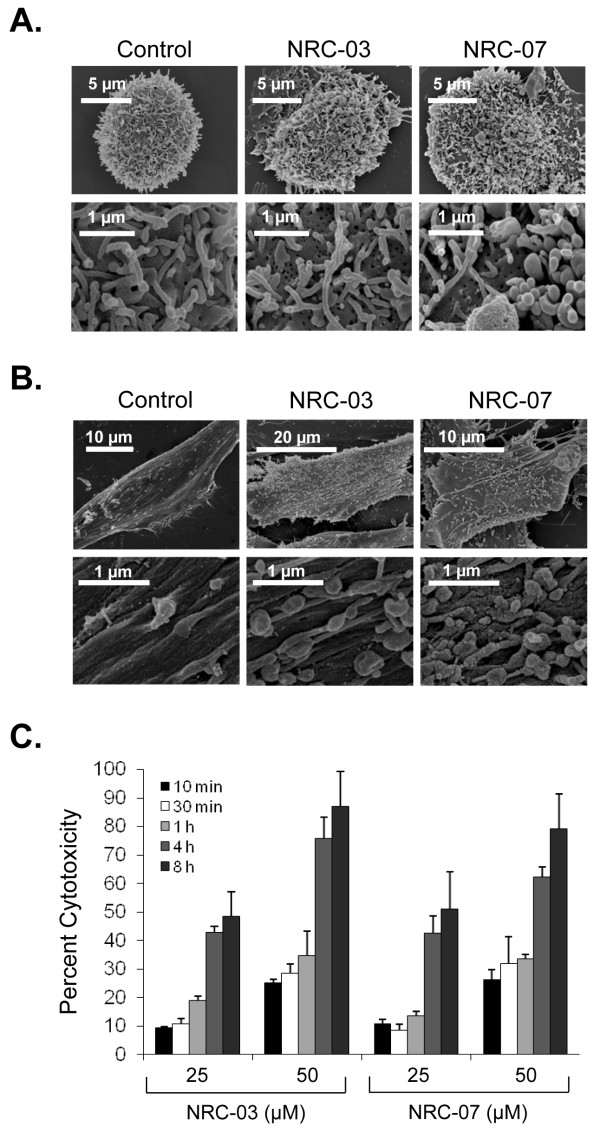
**NRC-03 and NRC-07 damage the cell membrane of breast cancer cells but not fibroblasts**. **(a) **MDA-MB-231 breast cancer cells or **(b) **normal dermal fibroblasts were cultured in the absence or presence of 50 μ*M *NRC-03 or NRC-07 for 30 minutes. Membrane damage was visualized with scanning electron microscopy. Data shown are from a representative experiment (*n *= 2). **(c) **MDA-MB-231 cells were cultured in the absence or presence of the indicated concentrations of NRC-03 and NRC-07. Cytotoxicity was measured with the LDH-release assay after 10 minutes, 30 minutes, and 1, 4, and 8 hours. Data are expressed as the mean ± SEM of three independent experiments.

**Figure 5:**
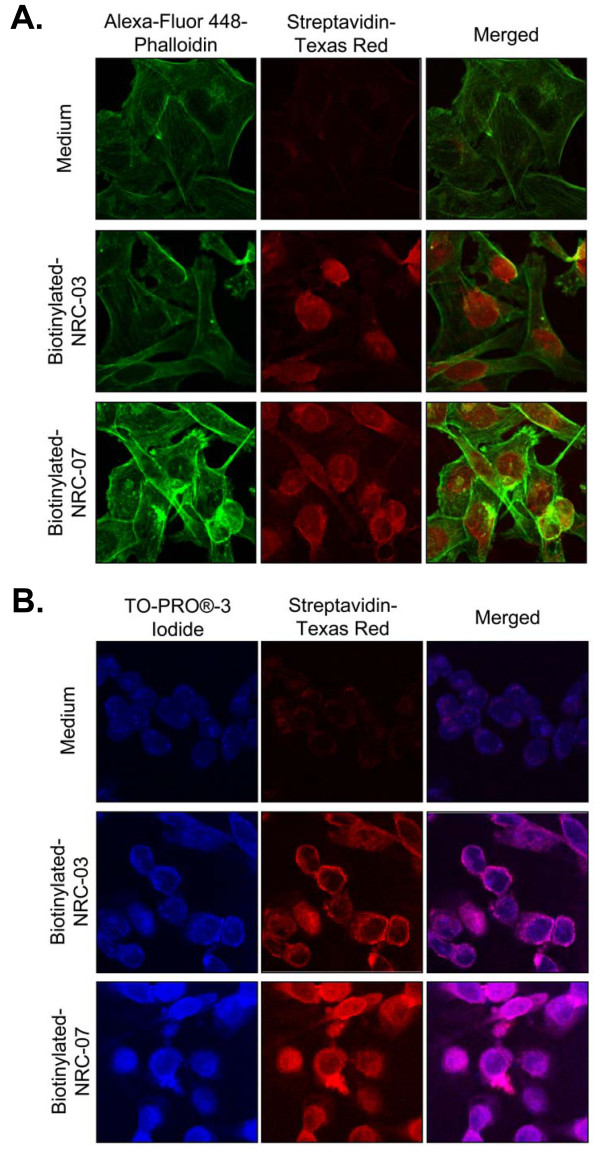
**NRC-03 and NRC-07 enter breast cancer cells and localize to the nucleus**. MDA-MB-231 breast cancer cells were cultured in the presence or absence of 50 μ*M *biotinylated-NRC-03, or -NRC-07 for 30 seconds. **(a) **The actin cytoskeleton and biotinylated-peptides were visualized with confocal microscopy (×1,000) by using Alexa-Fluor 488-phalloidin and Texas Red-conjugated streptavidin, respectively. **(b) **The nucleus and biotinylated-peptides were visualized with confocal microscopy (×1,000) by using TO-PRO-3 iodide and Texas Red-conjugated streptavidin, respectively. Images shown are from a representative experiment (*n *= 3).

CAPs kill cancer cells by causing significant and irreparable membrane damage and/or by inducing apoptosis [9]. Scanning electron microscopy was used to determine the effect of NRC-03 and NRC-07 on the membrane integrity of peptide-sensitive MDA-MB-231 breast cancer cells and peptide-resistant fibroblasts. Figure 4a shows that MDA-MB-231 cells exhibited substantial membrane damage after exposure to 50 μ*M *NRC-03 or NRC-07. Peptide-treated breast cancer cells had fewer microvilli, and those that remained were shrivelled in appearance. Numerous pores of various sizes were evident in nearly all peptide-treated breast cancer cells, which were swollen or had completely collapsed. These peptide-induced changes in morphology are consistent with a cytolytic mechanism of action. Normal fibroblasts remained intact after exposure to 50 μ*M *NRC-03 or NRC-07 (Figure 4b), although the number of microvilli appeared to increase. Consistent with a cytolytic effect of NRC-03 and NRC-07 on breast cancer cells, LDH was released in a time-dependent fashion by peptide-treated MDA-MB-231 cells, peaking after 4 hours of exposure (Figure 4c). Breast cancer cell-membrane disruption after NRC-03 and NRC-07 treatment was confirmed by propidium iodide uptake by MDA-MB-231 cells after 10-minute exposure to peptide (data not shown). Evidence of extensive CAP-mediated damage to the cell-membrane of breast cancer cells suggested that NRC-03 and/or NRC-07 might be able to enter the damaged cells. Fluorescence confocal microscopy revealed that biotinylated-NRC-03 and biotinylated NRC-07 rapidly entered the cytoplasm of peptide-treated MDA-MB-231 cells and appeared to localize to the nucleus (Figure 5a, b).

### NRC-03 and NRC-07 cause mitochondrial membrane damage and ROS production in breast cancer cells

**Figure 6:**
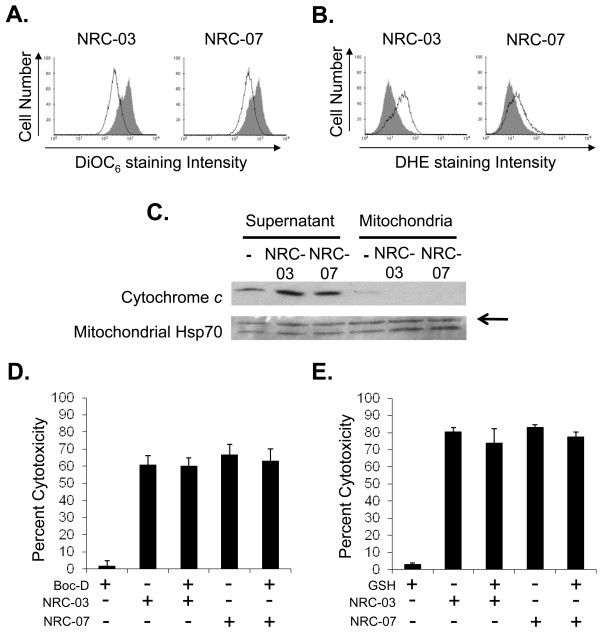
**NRC-03- and NRC-07-mediated cytotoxicity is associated with mitochondrial membrane damage and ROS generation**. MDA-MB-231 breast cancer cells were cultured in the absence or presence of NRC-03 or NRC-07 for 30 minutes. **(a) **DiOC6 or **(b) **DHE was added to cells to detect a loss of mitochondrial transmembrane potential and ROS generation, respectively. Solid peak, medium alone. Open peak, 50 μ*M *NRC-03 or NRC-07 treatment. **(c) **Mitochondria were isolated from MDA-MB-231 cells and treated with NRC-03 or NRC-07, as described earlier, for 10 minutes. Cytochrome *c *was detected in the supernatant and mitochondria fractions with Western blot analysis. Mitochondrial Hsp70 was detected to confirm equal protein loading. Data shown are from a representative experiment (*n *= 3). MDA-MB-231 cells were pretreated with **(d) **40 μ*M *Boc-D-FMK or **(e) **5 m*M *GSH before exposure to 50 μ*M *NRC-03 or NRC-07. Percentage cytotoxicity was assessed with MTT and acid phosphatase viability assays, respectively. Data shown are the mean ± SEM of three independent experiments and are not statistically significant (*p *> 0.05) in comparison with controls with the Bonferroni multiple comparisons test.

Because mitochondria carry a negative charge [31], NRC-03 and NRC-07 that enter breast cancer cells might target and damage mitochondria. DiOC_6 _staining showed that mitochondrial membrane integrity was lost after NRC-03 or NRC-07 treatment of MDA-MB-231 cells (Figure 6a). ROS generation was also detected by DHE staining within 30 minutes of MDA-MB-231 exposure to NRC-03 and NRC-07 (Figure 6b). Release of cytochrome *c *by isolated mitochondria that were treated with NRC-03 or NRC-07 (Figure 6c) confirmed mitochondrial membrane permeabilization by these peptides. Moreover, fluorescence confocal microscopy confirmed that biotinylated-NRC-03 and biotinylated-NRC-07 interacted with mitochondria of MDA-MB-231 cells (Additional file 2). Release of mitochondrial cytochrome *c *into the cytosolic compartment promotes apoptosis via caspase-9 activation [32]. However, neither caspase activation nor ROS production was required for NRC-03- or NRC-07-induced cytotoxicity, because pretreatment of MDA-MB-231 cells with the pancaspase inhibitor Boc-D-FMK (Figure 6d) or reduced GSH (Figure 6e) failed to protect the cells from peptide-induced cell death. Interestingly, TUNEL staining showed DNA fragmentation in NRC-07-treated MDA-MB-231 cells, although DNA appeared intact in NRC-03-treated MDA-MB-231 cells (Additional file 3).

Because autophagy has been reported sometimes to precede mitochondria-mediated apoptosis in cancer cells treated with a cytotoxic agent [33], we compared the effect of NRC-03 and NRC-07 on wild-type and autophagy-related gene 5 (ATG5)-deficient mouse embryo fibroblasts that are refractory to autophagy-like cell death [26]. However, no difference in sensitivity to killing by the CAPs was noted (Additional file 4), suggesting that NRC-03 and NRC-07 do not cause autophagy-like cell death.

### NRC-03 and NRC-07 inhibit breast cancer xenograft growth

**Figure 7:**
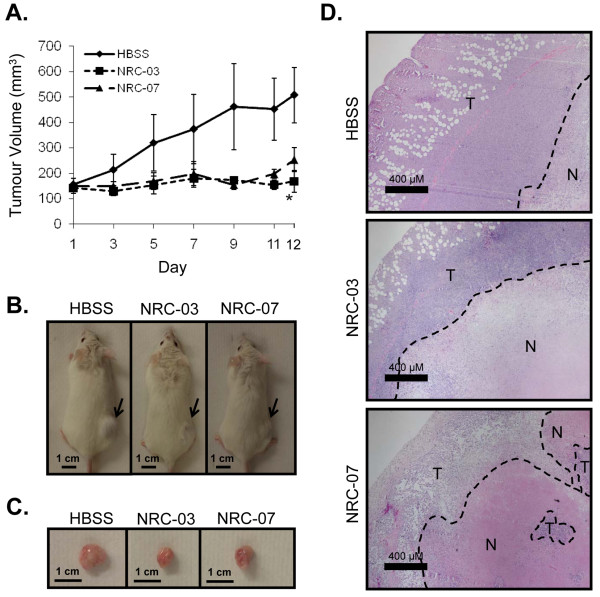
**NRC-03 and NRC-07 halt the growth of breast cancer xenografts in mice**. MDA-MB-231 breast cancer cells were implanted in the right hind flank of NOD SCID mice. Once tumors reached a volume at least 120 mm^3^, they were injected with the HBSS alone or with 0.5 mg NRC-03 or NRC-07 (in HBSS) on days 1, 3, and 5. **(a) **Tumor volume was determined on days 1, 3, 5, 7, 9, 11, and 12 after the start of peptide treatment. Data shown are the mean tumor volume ± SEM from three independent experiments (three mice per group conducted three times for a total of nine mice per treatment). Statistical significance was determined with the Bonferroni multiple comparisons test; *p < 0.05 compared with HBSS-treated animals. **(b) **On day 12, mice were killed and photographed, and **(c) **the tumors were excised and photographed **(d)**. Tumor sections were stained with hematoxylin and eosin, and photographed by using bright-field microscopy (×400). Scale bars indicate 400 μm. T, viable cells; N, necrotic cells. Representative images are shown.

Finally, we tested the *in vivo *activity of NRC-03 and NRC-07 in immune-deficient NOD SCID mice implanted with MDA-MB-231 breast cancer cells, which form subcutaneous tumors. Xenografted tumor-bearing mice received intratumoral injections of HBSS alone or 0.5 mg NRC-03 or NRC-07 on days 1, 3, and 5 once tumors reached a volume of at least 120 mm^3^. Tumor volume was then monitored over the next 12 days. As shown in Figure 7a, peptide-treated tumors failed to grow beyond their initial size after the start of treatment and, in the case of NRC-03-treated tumors, were significantly smaller (*p *< 0.05) than control tumors at day 12. NRC-07-treated tumors showed a similar trend, but the difference did not reach statistical significance. In contrast, tumors that were injected with the NRC-13 control peptide did not exhibit reduced growth (Additional file 5). NRC-03- and NRC-07-treated tumors also appeared to be smaller both before and after excision (Figure 7b, c). Hematoxylin-and-eosin staining showed that peptide-treated tumors contained a larger necrotic area (outlined by dashed lines) than did HBSS-treated tumors (Figure 7d), which is consistent with NRC-03- and NRC-07-mediated lysis of tumor cells. Intratumoral administration of the CAPs did not have any discernable adverse effects on the mice. Necropsies conducted on control and peptide-treated animals did not reveal any marked differences between treatment groups, nor were there significant differences in weight.

## Discussion

Chemotherapeutic drugs that are currently used in cancer treatment are limited by their nonspecific toxicity, inability to kill slow-growing and drug-resistant cancer cells, and potential to cause secondary malignancies [3,4,6]. These drawbacks have stimulated the search for novel anticancer agents that selectively kill cancer cells, including drug-resistant variants, regardless of their rate of growth. In this study, we showed for the first time that pleurocidin-family CAPs NRC-03 and NRC-07 are cytotoxic for multiple breast cancer cell lines, including SKBR3 cells that contain a 100% ALDEFLUOR-positive breast cancer stem cell population [34]. In addition, only 4-hour exposure to NRC-03 or NRC-07 was needed to reduce significantly the viability of paclitaxel-resistant MCF7-TX400 cells, indicating that these CAPs are able to kill drug-resistant breast cancer cells. NRC-03 and NRC-07 also killed cisplatin-resistant ovarian cancer cells (Hilchie and Hoskin, unpublished data), suggesting that these CAPs are able to kill cancer cells that develop chemoresistance by mechanisms other than increased expression of P-glycoprotein.

NRC-03 and NRC-07 did not substantially harm HUVECs or human fibroblasts at concentrations that were strongly cytolytic for breast cancer cells; however, HMECs were susceptible to killing by both peptides. Nevertheless, the nonspecific toxicity of systemic NRC-03 and NRC-07 might still be less than that of traditional chemotherapeutic agents. Moreover, the nonspecific toxicity of certain CAPs can be substantially reduced by the addition of cancer cell-targeting moieties [15,16,35]. In addition, CAPs can be modified for pH-dependent activation in the acidic tumor microenvironment by replacing lysine and/or arginine residues with histidine residues [36]. Importantly, neither NRC-03 nor NRC-07 exhibited hemolytic activity, which is a drawback that reduces the therapeutic utility of some other α-helical CAPs [23].

CAPs such as NRC-03 and NRC-07 that exhibit anticancer activity are believed to target cancer cells on the basis of charge rather than the rate of cell growth (9,11,12), which gives CAPs a completely different mechanism of action than that of conventional chemotherapeutic agents. NRC-03 and NRC-07 exhibited a 56- and 98-fold greater binding capacity, respectively, to breast cancer cells than to normal fibroblasts, even though the doubling time of the neoplastic epithelial cells was approximately the same as that of the untransformed fibroblasts. The potent cytolytic activity of NRC-03 and NRC-07 against slow-growing SKBR3 cells (~36-hour doubling time) suggests that these peptides may be effective against indolent tumors. Peptide binding to anionic heparan sulfate proteoglycans and chondroitin sulfate proteoglycans was involved in NRC-03- and NRC-07-mediated cytotoxicity; however, none of these molecules was exclusively necessary for target-cell death, suggesting that NRC-03 and NRC-07 interact with additional negatively-charged molecules on the surface of susceptible cells. In this regard, increased density of negatively-charged phosphatidylserine on the surface of cancer cells has been suggested to serve as a target for CAPs [37]. Because many different negatively-charged molecules contribute to the overall anionic charge of cancer cells that renders them susceptible to CAP-mediated killing, it is unlikely that breast cancer and other malignant cells will be able easily to acquire resistance to cytotoxic CAPs such as NRC-03 and NRC-07.

Electrostatic interactions between breast cancer cells and NRC-03 or NRC-07 resulted in severe damage to the cell membrane, as demonstrated with scanning electron microscopy, propidium iodide uptake, and the release of cellular LDH. Integration of bulky hydrophobic amino acids into the hydrophobic core of the target cell membrane and adoption of a stable amphipathic structure is believed to lead to pore formation by CAPs [9]. Interestingly, percentage cytotoxicity in LDH-release assays typically exceeded cytotoxicity measured by MTT assays, suggesting that the MTT assay underestimated the killing of breast cancer cells by NRC-03 and NRC-07. In addition, treatment with NRC-03 and NRC-07 caused mitochondrial transmembrane potential to be lost in breast cancer cells, as well as inducing ROS production, possibly as a result of the CAPs targeting and damaging mitochondria, because fluorescence confocal microscopy showed colocalization of peptides and mitochondria. Moreover, NRC-03 and NRC-07 were able to permeabilize preparations of isolated mitochondria. However, apoptosis and ROS generation associated with mitochondrial permeabilization was not required for NRC-03- and NRC-07-mediated cytotoxicity because the addition of a pancaspase inhibitor or reduced GSH failed to protect breast cancer cells from killing by the peptides. Nevertheless, NRC-07 was able to cause DNA fragmentation in breast cancer cells, as indicated by TUNEL staining. Interestingly, both NRC-03 and NRC-07 appeared to localize rapidly to the nucleus of peptide-treated breast cancer cells, possibly because of peptide interactions with anionic nucleic acids. We conclude that NRC-03 and NRC-07 directly kill breast cancer cells by a membranolytic mechanism, although we cannot rule out the possibility that NRC-03- and/or NRC-07-induced pore formation in mitochondria may contribute to cytotoxicity.

It is also conceivable that prolonged exposure to lower concentrations of NRC-03 and/or NRC-07 may cause transient cell-membrane damage and induce cell death by mitochondrial-dependent apoptosis or an inhibition of macromolecular synthesis. This dual effect of pleurocidin has been demonstrated in bacteria [38]; however, the impact of prolonged exposure to low concentrations of NRC-03 and NRC-07 on breast cancer cell viability has not yet been investigated.

Sublethal concentrations of NRC-03, and, to a lesser extent, NRC-07, significantly reduced the EC_50 _of cisplatin, leading us to conclude that NRC-03 and NRC-07 possess chemosensitizing properties. A membranolytic mechanism of action likely accounts for the observed ability of NRC-03 and NRC-07 to enhance the killing of breast cancer cells by cisplatin. In addition, nuclear localization of NRC-03 and NRC-07 is predicted to disrupt the nuclear membrane and allow easier access of cisplatin and other DNA-crosslinking agents to the nucleus. However, it is not yet known whether sublethal doses of NRC-03 and/or NRC-07 similarly enhance *in vivo *cytotoxicity mediated by chemotherapeutic drugs.

Unlike control breast cancer xenografts in NOD SCID mice, flank tumors that received intratumoral injections of NRC-03 or NRC-07 did not increase in size once peptide treatment was started, whereas tumors that were injected with a noncytotoxic control peptide grew at the same rate as HBSS-injected tumors. In addition, histologic analysis revealed that the necrotic core of peptide-treated tumors was larger than that of control tumors, which is consistent with the *in vitro *cytolytic activity of NRC-03 and NRC-07. Importantly, intratumoral delivery of NRC-03 and NRC-07 to mice did not have any noticeable adverse side-effects, indicating that NRC-03 and NRC-07 can be safely administered via intratumoral injection. Interestingly, Berge and colleagues [19] recently demonstrated that intratumoral injection of another lytic peptide stimulated a protective antitumor immune response in immune-competent mice as a result of peptide-mediated lysis of tumor cells providing an immunostimulatory "danger signal" to T cells. The cytolytic mechanism of action of NRC-03 and NRC-07 suggests that intratumoral administration of these peptides may also stimulate an antitumor immune response.

## Conclusions

Conventional chemotherapeutic drugs are limited by their lack of specificity for cancer cells and their inability to kill multidrug-resistant and slow-growing cancer cells. We have shown for the first time that the pleurocidin-family CAPs NRC-03 and NRC-07 are cytotoxic for multiple breast cancer cell lines, including MCF7-TX400 cells that overexpress P-glycoprotein, and slow-growing SKBR3 cells that contain a 100% ALDEFLUOR-positive breast cancer stem cell population. We established that NRC-03- and NRC-07-mediated cell death is initiated by peptide binding to negatively-charged molecules on the surface of breast cancer cells. NRC-03 also substantially reduces the EC_50 _of cisplatin, suggesting the possible use of NRC-03 as a chemosensitizing agent. Importantly, both NRC-03 and NRC-07 killed breast cancer cells grown in NOD SCID mice. These findings indicate that NRC-03 and NRC-07 have several advantages over conventional chemotherapeutic drugs and warrant further investigation as possible novel anticancer agents.

## Abbreviations

CAP: Cationic antimicrobial peptide; CFU: colony-forming units; DHE: dihydroethidium; DiOC_6_: 3,3'-dihexyloxacarbocyanine iodide; DMEM: Dulbecco's modified Eagle's medium; DMSO: dimethyl sulfoxide; FBS: fetal bovine serum; GSH: glutathione; HBSS: Hank's balanced salt solution; HEPES: 4-(2-hydroxyethyl)-1-piperazineethanesulfonic acid; HMEC: human mammary epithelial cell; HRP: horseradish peroxidise; HUVEC: human umbilical vein endothelial cell; mAb: monoclonal antibody; NOD SCID: non-obese diabetic severe combined immunodeficient; OSGE: *O*-sialoglycoprotein endopeptidase; ROS: reactive oxygen species.

## Competing interests

S Douglas has a patent filed in the United States in 2003 and in Europe in 2008.

## Authors' contributions

AH participated in study design, conducted the experiments, and drafted the manuscript. CD participated in the animal studies. DP performed MALDI-TOF mass spectrometry. AP and SD provided advice on the study design. DH conceived the study, participated in its design, and finalized the manuscript. All authors have read and approved the manuscript.

## Supplementary Material

Additional file 1 **NRC-03 and NRC-07 are susceptible to degradation by proteases**. (a) MDA-MB-231 cells cultured in the presence of 0.5, 2.5, and 5% FBS were exposed to 50 μ*M *NRC-03 or NRC-07. Cell viability was determined with MTT assay after 24 hour. Data shown are statistically significant by ANOVA (*p *< 0.05) and represent the mean of three independent experiments ± SEM. **(b) **The 50 μg of NRC-03 or NRC-07 was combined with 1 μg trypsin and incubated overnight at 37°C. Intact and/or fragmented peptides were detected with MALDI-TOF mass spectrometry. Data shown are from one experiment. Click here for file

Additional file 2 **NRC-03 and NRC-07 interact with mitochondria in breast cancer cells**. MDA-MB-231 breast cancer cells were cultured in the presence or absence of 50 μ*M *biotinylated-NRC-03 or biotinylated-NRC-07 for 30 seconds. Biotinylated peptides and mitochondria were visualized with confocal microscopy (×1,000) by using Texas Red-conjugated streptavidin and anti-mitochondrial Hsp70 mAb, respectively. Arrows point to sites of colocalization. Images shown are from a representative experiment (*n *= 3). Click here for file

Additional file 3 **NRC-07, but not NRC-03, causes DNA fragmentation in breast cancer cells**. MDA-MB-231 breast cancer cells were cultured in the presence or absence of 50 μ*M *NRC-03 or NRC-07 for 30 minutes. DNA fragmentation was detected with TUNEL staining that was visualized with fluorescence microscopy. Data shown are from a representative experiment (*n *= 3). Click here for file

Additional file 4 **NRC-03 and NRC-07 do not cause autophagy-like cell death**. ATG5^+/+ ^or ATG5 ^-/- ^mouse embryo fibroblasts (MEFs) were cultured in the presence or absence of 50 μ*M *NRC-03 or NRC-07. Cell viability was determined with MTT assay after 24 hours. No statistically significant difference (*p *> 0.05) was found between peptide-mediated killing of ATG5^+/+ ^or ATG5 ^-/- ^mouse embryo fibroblasts, as determined with the Student *t *test. Data shown are the mean of at least three independent experiments ± SEM. Click here for file

Additional file 5 **The noncytotoxic control peptide NRC-13 does not have antitumor activity**. MDA-MB-231 breast cancer cells were implanted in the hind flanks of NOD SCID mice. Once tumors reached a volume at least 120 mm^3^, they were injected with HBSS alone or with 0.5 mg NRC-03 or NRC-13 (in HBSS) on days 1, 3, and 5. Tumor volumes were determined on days 1, 3, 5, 7, 9, 11, and 12 after the start of peptide treatment. Data shown are the mean of five animals ± SD. Statistical significance was determined with the Bonferroni multiple comparisons test; *p < 0.05 compared with HBSS-treated animals. Click here for file
