# Supplementary figures and images for: Pleurocidin-family cationic antimicrobial peptides are cytolytic for breast carcinoma cells and prevent growth of tumor xenografts
Source: Breast Cancer Res. 2011 Oct 24;13(5):R102. doi: 10.1186/bcr3043 (PMC3262215; doi:10.1186/bcr3043)

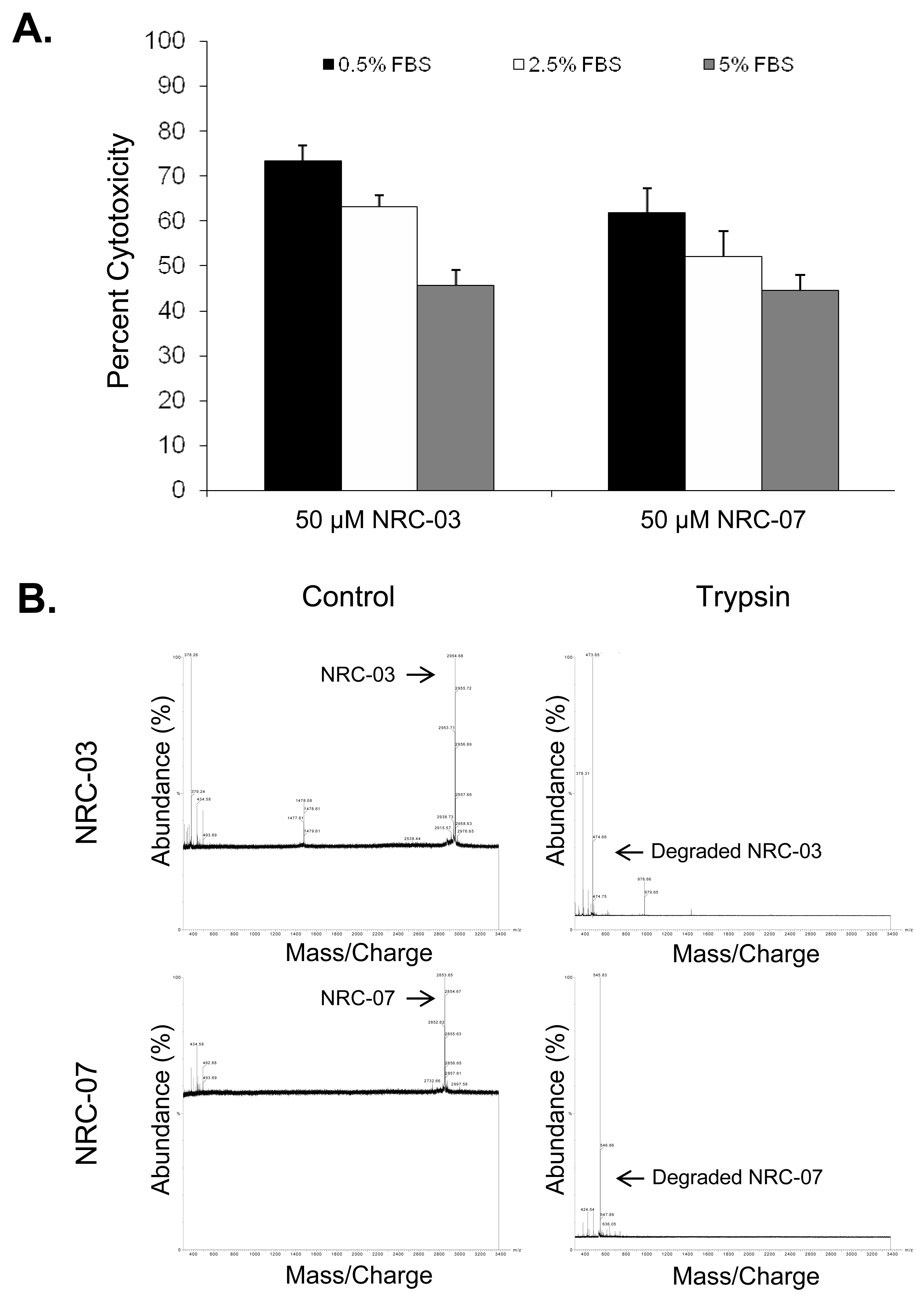

Supplement: Additional file 1 — NRC-03 and NRC-07 are susceptible to degradation by proteases. (a) MDA-MB-231 cells cultured in the presence of 0.5, 2.5, and 5% FBS were exposed to 50 μM NRC-03 or NRC-07. Cell viability was determined with MTT assay after 24 hour. Data shown are statistically significant by ANOVA (p < 0.05) and represent the mean of three independent experiments ± SEM. (b) The 50 μg of NRC-03 or NRC-07 was combined with 1 μg trypsin and incubated overnight at 37°C. Intact and/or fragmented peptides were detected with MALDI-TOF mass spectrometry. Data shown are from one experiment. [file bcr3043-S1.TIFF]

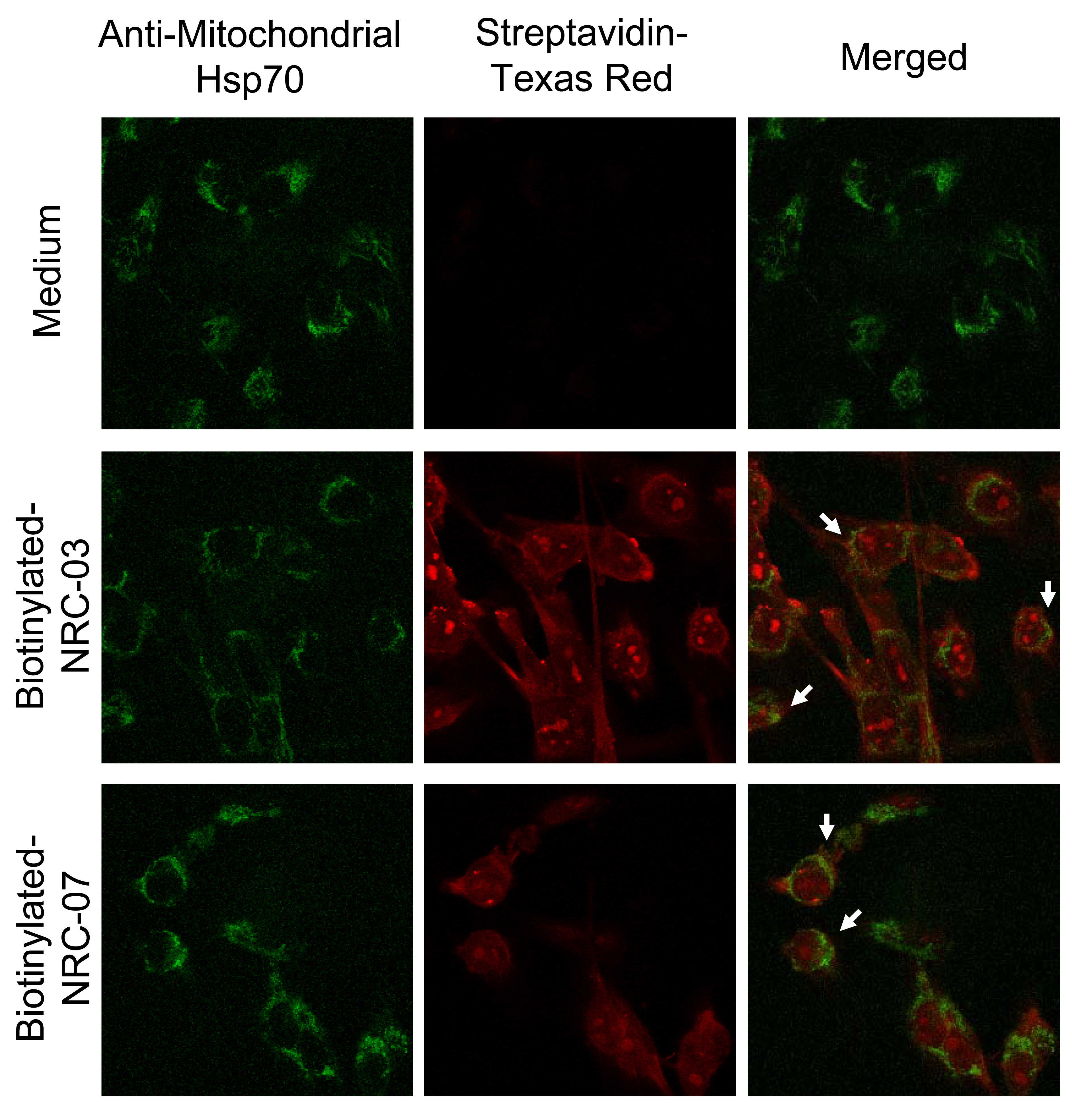

Supplement: Additional file 2 — NRC-03 and NRC-07 interact with mitochondria in breast cancer cells. MDA-MB-231 breast cancer cells were cultured in the presence or absence of 50 μM biotinylated-NRC-03 or biotinylated-NRC-07 for 30 seconds. Biotinylated peptides and mitochondria were visualized with confocal microscopy (×1,000) by using Texas Red-conjugated streptavidin and anti-mitochondrial Hsp70 mAb, respectively. Arrows point to sites of colocalization. Images shown are from a representative experiment (n = 3). [file bcr3043-S2.TIFF]

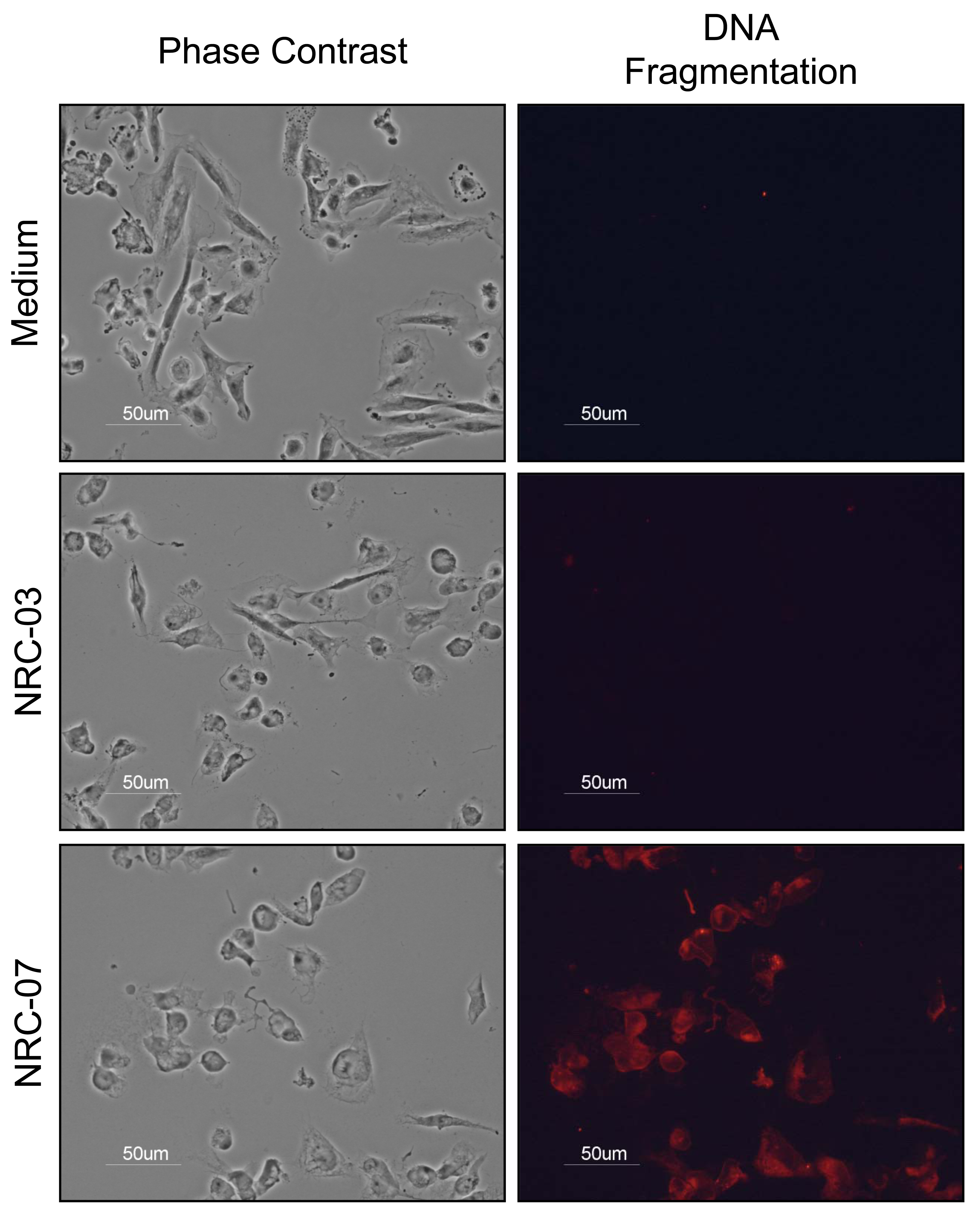

Supplement: Additional file 3 — NRC-07, but not NRC-03, causes DNA fragmentation in breast cancer cells. MDA-MB-231 breast cancer cells were cultured in the presence or absence of 50 μM NRC-03 or NRC-07 for 30 minutes. DNA fragmentation was detected with TUNEL staining that was visualized with fluorescence microscopy. Data shown are from a representative experiment (n = 3). [file bcr3043-S3.TIFF]

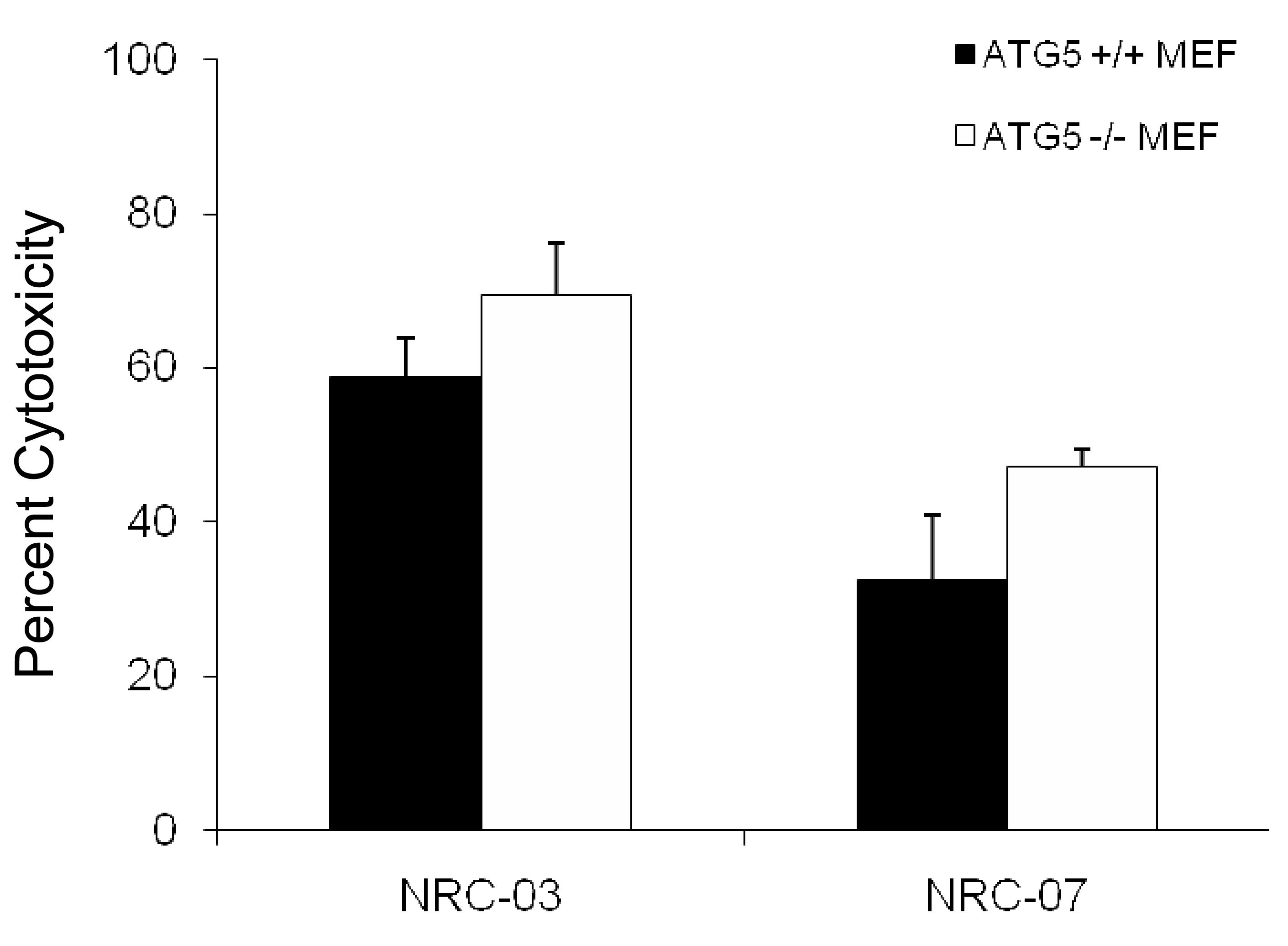

Supplement: Additional file 4 — NRC-03 and NRC-07 do not cause autophagy-like cell death. ATG5+/+ or ATG5 -/- mouse embryo fibroblasts (MEFs) were cultured in the presence or absence of 50 μM NRC-03 or NRC-07. Cell viability was determined with MTT assay after 24 hours. No statistically significant difference (p > 0.05) was found between peptide-mediated killing of ATG5+/+ or ATG5 -/- mouse embryo fibroblasts, as determined with the Student t test. Data shown are the mean of at least three independent experiments ± SEM. [file bcr3043-S4.TIFF]

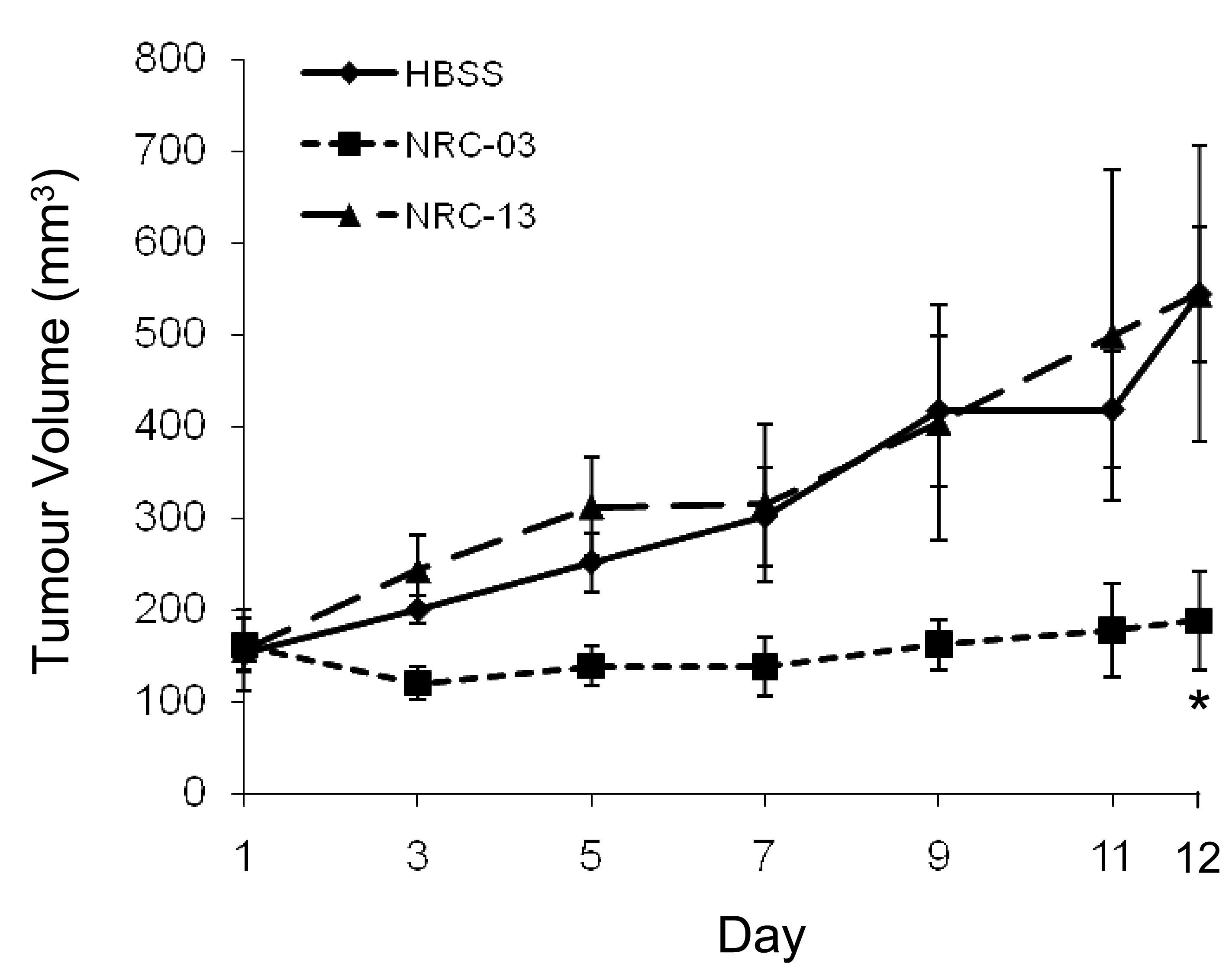

Supplement: Additional file 5 — The noncytotoxic control peptide NRC-13 does not have antitumor activity. MDA-MB-231 breast cancer cells were implanted in the hind flanks of NOD SCID mice. Once tumors reached a volume at least 120 mm3, they were injected with HBSS alone or with 0.5 mg NRC-03 or NRC-13 (in HBSS) on days 1, 3, and 5. Tumor volumes were determined on days 1, 3, 5, 7, 9, 11, and 12 after the start of peptide treatment. Data shown are the mean of five animals ± SD. Statistical significance was determined with the Bonferroni multiple comparisons test; *p < 0.05 compared with HBSS-treated animals. [file bcr3043-S5.TIFF]
